# Long-Term Evolution of Post-COVID-19 Echocardiographic Parameters in Patients with Chronic Kidney Disease: A Prospective Comparative Observational Study

**DOI:** 10.3390/jcm14061823

**Published:** 2025-03-08

**Authors:** Laura Vasiliu, Anca Diaconu, Mihai Onofriescu, Gianina Dodi, Alexandra Covic, Alexandra E. Avanu, Luminita Voroneanu, Vlad Vasiliu, Mehmet Kanbay, Radu A. Sascău, Cristian Stătescu, Adrian C. Covic

**Affiliations:** 1 Faculty of Medicine, “Grigore T. Popa” University of Medicine, 700115 Iasi, Romania; mihai.onofriescu@gmail.com (M.O.); gianina.dodi@umfiasi.ro (G.D.); covic.alexandra@yahoo.com (A.C.); avanualexandra@yahoo.com (A.E.A.); lumivoro@yahoo.com (L.V.); cstatescu@gmail.com (C.S.); accovic@gmail.com (A.C.C.); 2 Cardiology Department, Cardiovascular Diseases Institute “Prof. Dr. George I. M. Georgescu”, 700503 Iasi, Romania; anca.a.diaconu@gmail.com; 3 Department of Medicine, Division of Nephrology, Koc University School of Medicine, Istanbul 34450, Turkey; drkanbay@yahoo.com; 4 Academy of Romanian Scientists, 050044 Bucharest, Romania; 5 Academy of Medical Sciences, 020125 Bucharest, Romania

**Keywords:** COVID-19, RV systolic function, myocardial performance, end stage renal disease

## Abstract

**Background/Objectives**: Severe acute respiratory syndrome coronavirus-2 (SARS-CoV-2) has caused post-acute sequelae, especially for people with pre-existing conditions, including chronic kidney disease (CKD), which may impact the cardiovascular system. Yet, despite the preliminary description of the general population’s long-COVID-19 consequences, data on CKD patients is scarce. The aim of this study was to investigate the longitudinal effects of COVID-19 on echocardiographic parameters of cardiac function and on cardiac biomarkers in patients with CKD. **Methods**: A total of 163 patients were included in this observational prospective trial (listed under NCT05125913 code): 88 in the COVID-19 group and 75 in the control group. The serial echocardiographic characteristics in patients who survived beyond one year, focused on left and right ventricular systolic function, together with cardiac biomarkers evolution, were compared between the two groups. **Results**: At baseline, there were no significant differences in left ventricular (LV) function parameters, except for a higher Tei Index in the COVID-19 group (*p* < 0.01). Right ventricular (RV) systolic dysfunction was more frequent in the COVID-19 group, with worse fractional area change (FAC) (*p* = 0.01), RV free wall longitudinal strain (RVFWLS) (*p* = 0.01), and RV Tei Index (*p* = 0.01). Over time, the control group showed a decline in LV ejection fraction (EF), while the COVID-19 group slightly improved. RV global systolic function was better preserved in the COVID-19 group. To the best of our knowledge, this is the first study that demonstrates a statistically significant increase in LAVi in patients with COVID-19. **Conclusions**: Prior COVID-19 infection influenced the trajectory of LV and RV function in CKD patients over 12 months, suggesting potential transient myocardial adaptations. While overall cardiac function did not differ significantly between groups, COVID-19 survivors exhibited better preservation of some ventricular function parameters.

## 1. Introduction

In an unprecedented worldwide health situation of the 21st century, the COVID-19 pandemic, caused by the SARS-CoV-2 virus, challenged cardiologists to identify and treat a series of cardiovascular (CV) manifestations associated with respiratory distress. Since the beginning of the pandemic, numerous studies have investigated the echocardiographic profile of COVID-19 patients, first to provide a more accurate description of the infection’s impact on the heart, followed by the attempt to find correlations with other biological parameters, and ultimately to develop prognostic prediction models. In a cohort of 1216 confirmed COVID-19 patients who underwent echocardiographic evaluation, 37.4% had LV systolic dysfunction, and 14.3% presented with biventricular systolic impairment. Additionally, approximately half of the patients without pre-existing CV conditions displayed echocardiographic changes, including alterations in LV function [1]. Biventricular dilation and systolic dysfunction are more common after COVID-19 among patients who had increased cardiac biomarkers during the acute setting [2]. Regardless of the presence of structural heart disease, even mild forms of COVID-19 infection appear to be associated with changes in cardiac function within 3 weeks to 3 months after recovery—more specifically, higher global longitudinal strain (GLS), systolic pulmonary artery pressure, lower septal tissue velocities, and LV EF and at least moderate diastolic dysfunction [3].

Serial echocardiographic examinations performed in patients without a history of structural cardiac disease described initial cardiac changes in the acute setting due to adaptative mechanisms to increased hemodynamic stress, followed by a gradual improvement in parameters of the left atrial and RV function, as well as a decrease in parameters defining LV hypertrophy, over a 3-month follow-up. At one-year follow-up, an improvement of biventricular deformation parameters, a decrease in RV diameter, right atrial volume, and transtricuspidian regurgitant gradient were observed [4]. Elevated levels of cardiac biomarkers, such as troponin (Tn) and brain natriuretic peptide (BNP), were independent predictors of echocardiographic abnormalities, primarily represented by reduced cardiac output and increased LV filling pressures [5].

It is well-acknowledged that the CKD population suffers cardiac remodeling, mainly defined by increased LV hypertrophy, which is found in 14% to 74% of the patients [6,7]. As CKD progresses, a decline in LV systolic function, either defined by a low EF or a reduced GLS, as well as in diastolic function, is observed [6,8,9]. Indexed left atrial volume (LAVi), an indicator of diastolic dysfunction, is significantly higher in CKD patients [10]. More than two-thirds of the patients on dialysis have at least one echocardiographic abnormality, most frequently LV hypertrophy and left atrial enlargement [11]. Though RV systolic dysfunction is only found in 5% of patients on dialysis, it is the echocardiographic finding with the strongest association with MACE [11].

Long-COVID-19, defined as symptoms persisting for 3 months or more, leads to severe and prolonged functional impairment in multiple organs, as stated by Greenhalgh et al. in a clinical update published in August 2024 [12]. Yet, despite thousands of academic papers mentioning long- or post-COVID-19 sequelae, just a few studies examine the long-term health effects of COVID-19 on CKD patients. Post-acute sequelae of SARS-CoV-2 on individuals with CKD who were infected with COVID-19 were investigated by Filev et al. over a 3-year period. The collected data, namely clinical records (creatinine, eGFR, urea, D-dimer, leucocytes, and C-reactive protein) and patient-reported outcomes (fatigue, cognitive dysfunction, chest pain, and sleep disturbances) from 51 CKD and 46 non-CKD patients who survived COVID-19 infection, indicated no permanent impairment of renal function or progression of CKD. Only 23 patients were evaluated by echocardiography, with 5 patients further facing coronary angiography; however, no data on cardiac sequels were detected [13]. The findings of the Mahalingasivam et al. study suggested an association between COVID-19 and accelerated kidney function decline determined by the mean annual change in eGFR before vs. after each infection on 94 (79–107) mL/min/1.73 m^2^ median baseline eGFR patients [14]. However, the CKD population remains largely understudied, both in general and in the context of COVID-19, regarding cardiac function and structure. To address this gap, we conducted the first prospective cohort observational study on long-COVID-19 cardiac conditions in the context of CKD patients over a 12-month period. Therefore, the goal of this study was to examine the progression of left and right ventricular function along with cardiac biomarkers association on CKD patients, both with and without COVID-19 infection beyond 1-year survival.

## 2. Materials and Methods

### 2.1. Study Population

The study was conducted at Dr. C.I. Parhon University Hospital in Iasi, Romania, and additionally included patients from North-East Romania and from dialysis units affiliated with the hospital.

The study population was divided into two groups. The first group included patients over 18 years old with CKD stages 3–5, patients on dialysis, and kidney transplant (KTx) recipients with confirmed COVID-19, at minimum 2 weeks after the confirmed test, following the resolution of the active infection. The control group included patients with similar CKD stages without confirmed SARS-CoV-2 infection. The exclusion criteria were the presence of a prior diagnosis of pulmonary fibrosis, pneumectomy or massive pleural effusion, active malignancies, pregnancy, active systemic infections, and congenital heart disease. The enrolled patients provided written informed consent previously approved by the Ethical Committee of Grigore T. Popa University of Medicine and Pharmacy of Iasi (no. 110/2021).

### 2.2. Study Design

The study is part of the CARDIO SCARS IN CKD multi-center observational, prospective trial listed in the ClinicalTrial.gov database under the identifier NCT05125913, and its protocol was previously published [15]. Briefly, this trial was designed to evaluate CV risk in all consecutive patients with CKD (stages 3 to 5), those on dialysis, and KTx recipients following SARS-CoV-2 infection compared to a control group of patients. COVID-19 was diagnosed in accordance with the interim guidelines of the WHO, using the reverse transcriptase polymerase chain reaction (RT-PCR) assay on nasal or throat swabs [16]. Data collection took place between October 2021 and October 2022, with a follow-up period extending until October 2023. Eligible participants were identified through electronic health records and direct physician referrals.

After confirming eligibility, participants attended a baseline assessment, including a detailed interview and completion of the trial datasheet. Informed consent was obtained, and afterward, they proceeded with the study interventions. The participants continued their usual medical care and received copies of their records after each visit, available to their physician if needed. All dialysis patients underwent hemodialysis therapy three times per week and were evaluated on the second day after a dialysis session, during the short interdialytic interval.

### 2.3. Demographic and Echocardiography Parameters

At baseline, various demographic parameters were recorded, including age, sex, height, body weight, CKD etiology, dialysis/KTx vintage, smoking status, alcohol consumption history, diabetes, heart failure, coronary heart disease, chronic pulmonary disease, and chronic medication use. Additionally, COVID-19-related data were collected, such as the diagnosis date, disease severity, and the need for hospital admission, oxygen therapy, or other specific treatments.

Patients underwent clinical and echocardiographic evaluation at baseline, 6 months, and 12 months. Repeated assessments were performed to monitor changes over time, and only patients who survived at each respective time point were re-evaluated. All echocardiographic evaluations were conducted in accordance with the guidelines of the American Society of Echocardiography and the European Association of Cardiovascular Imaging [17], using the Philips CX50 ultrasound system (Andover, MA, USA) and QLAB 7.1 software (Andover, MA, USA).

All patients underwent a comprehensive echocardiographic evaluation, which assessed both cardiac anatomy (including diameters, volumes, and mass) and function, specifically the systolic performance of the left and right ventricles. Additionally, LV diastolic function and pulmonary hypertension parameters were reported. For this analysis, we focused on key parameters: EF, Tei index and GLS for LV systolic function, fractional area change (FAC), tricuspid annular longitudinal excursion (TAPSE), tricuspid S’ velocity, Tei index and right ventricular free wall longitudinal strain (RVFWLS) for RV systolic function. The LV EF was calculated using Simpson’s biplane method, while GLS was determined from three standard views (apical four-chamber, apical two-chamber, and apical three-chamber) using QLAB 7.1 software (Andover, MA, USA). High-quality cine loops with a high frame rate were utilized, with the region of interest (ROI) automatically estimated and manually adjusted to fit the LV wall thickness. The GLS was then calculated, generating a bull’s-eye plot. The mean value reported by two observers was used, with a normal GLS value defined as <−18%. FAC was determined by tracing the RV end-diastolic and end-systolic areas, while tricuspid S’ velocity was measured using tissue Doppler. TAPSE was measured by M-mode echocardiography. RVFWLS was calculated from a modified apical four-chamber view, also using QLAB 7.1 software, with the ROI manually adjusted to fit the RV wall thickness. The longitudinal strain was then calculated as the average value of the three segments of the right ventricular free wall, with a normal RVFWLS value considered to be <−23%. Tei index was measured using tissue Doppler for both left and right ventricles.

The serum concentration of Tn I (cTnI ELISA kit, cat. no. ABIN6730900, antibodies-online Inc., Aachen, Germany) and NT-ProBNP (NT-ProBNP ELISA kit, cat. no. ABIN368630, antibodies-online Inc., Aachen, Germany) were determined by sandwich enzyme-linked immunosorbent assay (ELISA). Blood samples were collected in gel and clot activator tubes during each clinical examination (at baseline, 6 and 12 months). Immediately after coagulation, the serum was separated by centrifugation (5 min at 4500 rpm) and stored in 0.5 mL aliquot tubes at −80 °C for subsequent ELISA. The assays were performed according to the manufacturer’s protocol. Briefly, 100 µL of sample (dilution factor 10) and the corresponding standards were added into appropriate wells and incubated for 1 h for TnI and 2 h for NT-ProBNP at 37 °C (on Matrix Orbital Delta plus IKA, Staufen, Germany). Then, 100 µL of biotin–antibody solution was added and incubated for 1 h at 37 °C, followed by washing and horseradish peroxidase (HRP)–avidin conjugate solution (100 µL) addition and incubation of 30 min for TnI and 1 h for NT-ProBNP. The washing procedure removed the unbound HRP-avidin, and 90 µL of TMB (3,3′, 5, 5′-Tetramethylbenzidine) substrate was added and incubated for another 25 min. The blue color was changed to yellow with a 50 µL add-on Stop solution. The optical density (OD) was determined at 450 nm using the Tecan Sunrise Plate Reader, Switzerland, and Magellan v. 7.2 software. All measurements were carried out in triplicate. The TnI and NT-ProBNP concentrations were determined using the standard curve generated through MyAssays online v. R10.2 software (regression analysis with four-parameter logistic) by plotting the mean OD values and concentration of each standard (detection range of 31.2–2000 pg/mL for cTnI and 31.2–20 ng/mL for NT-ProBNP). The inter-assay and intra-assay variances were 12% and 10% for both kit types.

### 2.4. Statistical Analysis

Statistical analysis was performed using IBM SPSS Statistics 26.0. Data were assessed for normality using the Shapiro–Wilk test and the assumption of sphericity was evaluated using Mauchly’s test. If sphericity was violated, the Greenhouse–Geisser or Huynh–Feldt correction was applied to adjust the degrees of freedom.

Continuous variables were presented as mean ± standard deviation (SD) and compared using independent samples *t*-test. Additionally, the median and interquartile range (IQR) were reported for non-normally distributed data. For non-normally distributed data, differences between two independent groups were assessed using the Mann–Whitney U test. Categorical variables were presented as frequencies and percentages and compared using the Chi-square test. Spearman’s rank-order correlation (ρ) was used to assess the strength and direction of monotonic relationships between variables.

A repeated-measures analysis of variance (rANOVA) was conducted to evaluate the longitudinal changes in echocardiographic parameters over three time points (baseline, 6 months, and 12 months) and to assess the interaction between time and COVID-19 status.

- Within-subjects factor: Time (3 levels);
- Between-subjects factor: COVID-19 status (2 levels);
- Dependent variables: LV EF, LV Tei Index, FAC, TAPSE, RVFWLS, RV Tei Index.

Effect sizes were reported using partial eta squared (η^2^). Post hoc pairwise comparisons with Bonferroni correction were applied for significant findings.

Since missing data were primarily due to mortality, imputation was not performed, and a complete case analysis was conducted, including only patients with available echocardiographic and biomarker data at each time point.

A *p*-value of <0.05 was considered statistically significant for all comparisons.

## 3. Results

### 3.1. Baseline Demographic and Clinical Characteristics

A total of 206 patients were included in this study: 121 in the COVID-19 group and 85 in the control group. As expected from previous reports, COVID-19 patients who were significantly older (*p* = 0.04) had a higher prevalence of diabetes (*p* = 0.01), heart failure (*p* = 0.01), and ischemic heart disease (*p* = 0.01). The average time between COVID-19 diagnosis and the baseline evaluation was 1.06 ± 0.67 months.

The dialysis group comprised 134 patients, with 79 in the COVID-19 group and 55 in the control group. The COVID-19 group had a mean dialysis vintage of 57.59 ± 64.07 months, while the control group had a mean dialysis vintage of 67.56 ± 79.47 months. The CKD group, including both CKD patients and KTx patients, consisted of 72 individuals, with 42 in the COVID-19 group and 30 in the control group. The demographics of the study population are detailed in Table 1.

### 3.2. Baseline Echocardiographic Characteristics

The prevalence of LV systolic dysfunction was higher in the COVID-19 group than in the control group, with rates of 45.5% vs. 34.1%, respectively. However, this difference was not statistically significant (*p* = 0.10). On the other hand, RV systolic dysfunction was significantly more prevalent in the COVID-19 group than in the control group: 30.6% vs. 17.6%, *p* = 0.03. There were no significant differences regarding diastolic dysfunction between COVID-19 and control groups: 19.8% vs. 12.9%, *p* = 0.19.

At baseline, there was no significant difference in LV EF between groups (55.52 ± 10.15% vs. 57.36 ± 8.77%, *p* = 0.19). The LV GLS had similar values between groups (−16.86 ± 4.44% vs. −17.72 ± 2.81%, *p* = 0.12). The LV Tei Index was significantly higher in COVID-19 patients (0.60 ± 0.16 vs. 0.50 ± 0.12, *p* < 0.01).

Patients from the COVID-19 group had a more impaired RV global systolic function when compared to the control group, illustrated by a significantly lower FAC (40.56 ± 10.82% vs. 45.02 ± 8.06%, *p* = 0.01). In addition, COVID-19 patients had worse RV longitudinal systolic dysfunction, expressed by lower RVFWLS (−18.01 ± 5.27% vs. −20.46 ± 2.93%, *p* = 0.01). The RV Tei Index was significantly higher in COVID-19 patients (0.60 ± 0.17 vs. 0.54 ± 0.16, *p* = 0.01). There was no significant difference in tricuspid S’ velocity between the two groups (13.12 ± 2.91 cm/s vs. 12.76 ± 2.93 cm/s, *p*= 0.38). TAPSE was also similar between groups (21.61 ± 4.81 mm vs. 22.17 ± 4.64 mm, *p* = 0.56). The proximal RVOT diameter was higher in the COVID-19 group (34.57 ± 5.00 mm vs. 32.94 ± 4.49 mm, *p* = 0.01) but without significant differences in terms of pulmonary acceleration time values (96.10 ± 26.17 ms vs. 100.40 ± 25.56 ms, *p* = 0.24).

Finally, COVID-19 patients showed marginally higher LAVi (43.17 ± 17.84 mL/m^2^ vs. 39.07 ± 16.22 mL/m^2^, *p* = 0.06), but there were no significant differences in LV filling pressures (11.19 ± 4.29 vs. 10.68 ± 4.89, *p* = 0.24) (Table 2).

### 3.3. Cardiac Biomarkers

At baseline, cardiac TnI levels in all patients were significantly elevated in the COVID-19 group compared to the control group (59.93 ± 69.28 pg/mL vs. 13.58 ± 13.99 pg/mL, *p* < 0.01). Similar results were observed when baseline values were calculated exclusively for the patients who survived. This significant difference persisted at 6 months, but by 12 months, the levels between the two groups were no longer significantly different.

There were no significant differences between COVID-19 and the control group at baseline in all patients in NT-proBNP values (4.82 ± 5.79 ng/mL vs. 3.21 ± 3.31 ng/mL, *p* = 0.28). This also applied to the patients who survived. However, over time, significant differences emerged, with COVID-19 patients having higher NT-proBNP values at both 6 months and 12 months.

The values of serial biomarker evaluations are detailed in Table 3.

### 3.4. Follow-Up Echocardiography

During the follow-up period, a total of 54 deaths were recorded: 40 in the COVID-19 group (5 among CKD patients and 35 among dialysis patients) and 14 in the control group (2 among CKD patients and 12 among dialysis patients). Of these, 11 patients passed away after a year. As a result, serial echocardiographic evaluations at 6 and 12 months were conducted for 163 patients, comprising 88 from the COVID-19 group and 75 from the control group.

The main echocardiographic parameters for LV and RV systolic function at baseline for the patients who survived are summarized in Table 4.

When serial echocardiographic evaluations were performed at 6 and 12 months after COVID-19, the baseline differences between standard indices for left and right ventricular systolic dysfunction lost their statistical significance (Table 5).

A repeated-measures ANOVA was conducted to examine the effect of time and COVID-19 status on LV EF. Time had a significant effect, as LV EF changed across time (*p* = 0.03). There was a significant effect between time and COVID-19, suggesting that the pattern of LV EF change differed between groups (*p* = 0.01). Post hoc contrasts revealed a significant linear effect of Time (*p* = 0.01) and a quadratic interaction effect (*p* = 0.01), indicating a more complex trajectory of LV EF for the COVID-19 group. The between-subjects effect of COVID-19 alone was non-significant (*p* = 0.95), suggesting that COVID-19 status did not independently impact overall LV EF levels.

**Figure 1:**
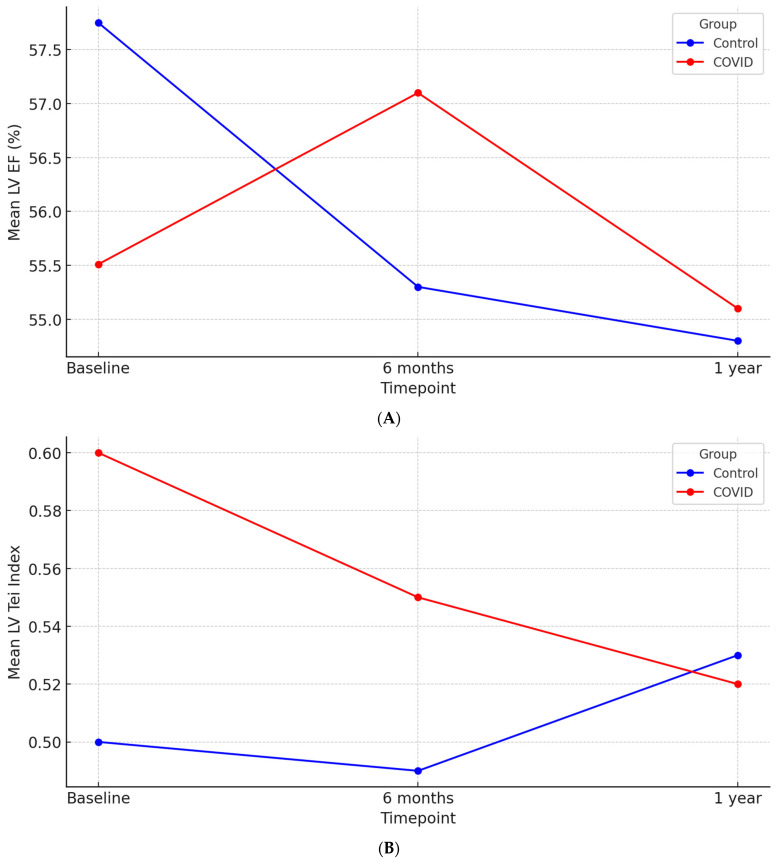
(**A**) Evolution of LV EF over time stratified by COVID-19 status. (**B**) Evolution of LV Tei Index over time stratified by COVID-19 status.

At 6 months, the LV EF in the control group decreased by 2.43% from baseline, while the COVID-19 group showed an improvement of 1.56% (ΔLV EF: −2.43 ± 7.48% vs. ΔLV EF: 1.56 ± 8.46%; *p* < 0.01). At 12 months, the mean absolute difference in LV EF from baseline was −2.91 ± 8.59% in the control group and −0.39 ± 8.68% in the COVID-19 group (*p* = 0.06), further illustrating the differential trajectory of LV EF over time (Figure 1A).

A repeated-measures ANOVA was conducted to examine the effect of time and COVID-19 status on the LV Tei Index. A significant effect of time was found, indicating that the LV Tei Index changed over time (*p* = 0.03). There was a significant effect between time and COVID-19 (*p* < 0.01), suggesting that the pattern of change over time differed based on COVID-19 status. The between-subjects effect of COVID-19 was significant (*p* = 0.01), and a significant linear effect of time was detected (*p* = 0.04), suggesting that COVID-19 influences the trajectory of the LV Tei Index over time (Figure 1B).

Analyzing mean differences from baseline, at 6 months, the COVID-19 group showed a significant improvement in the LV Tei Index compared to the control group (∆LV Tei Index −0.04 ± 0.19 vs. −0.00 ± 0.11, *p* = 0.05). This trend persisted at 12 months, with the COVID-19 group maintaining improved values (∆LV Tei Index −0.03 ± 0.18) while the control group exhibited an increase (0.04 ± 0.10, *p* = 0.01).

A repeated-measures ANOVA was conducted to examine the effect of time and COVID-19 status on FAC. Time had a significant effect (*p* = 0.02), indicating changes in FAC values over time. The time and COVID-19 interaction was not significant (*p* = 0.05), meaning the pattern of change over time was similar between groups. COVID-19 did not have a significant effect on overall scores (*p* = 0.09). The linear trend over time was significant (*p* = 0.04), suggesting an increasing or decreasing trajectory. These results suggest that FAC values change over time, but COVID-19 status does not significantly impact these changes.

**Figure 2:**
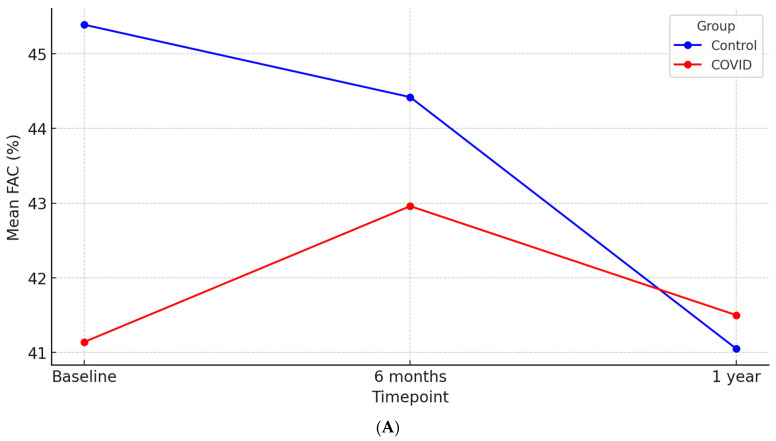

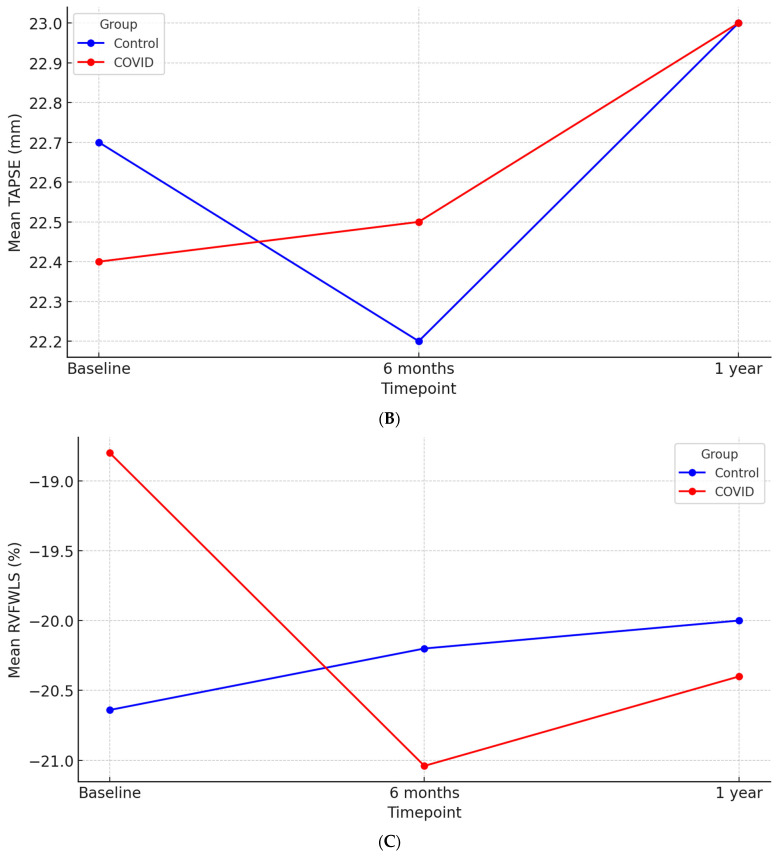

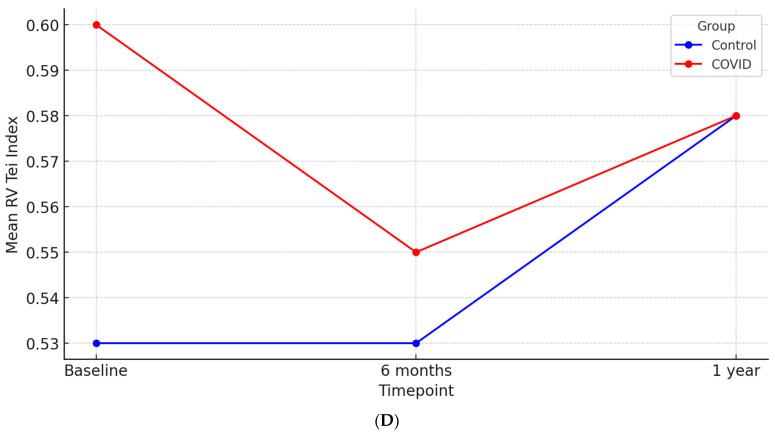
(**A**) Evolution of FAC over time stratified by COVID-19 status. (**B**) Evolution of TAPSE over time stratified by COVID-19 status. (**C**) Evolution of RVFWLS over time stratified by COVID-19 status. (**D**) Evolution of RV Tei Index over time stratified by COVID-19 status.

When examining mean differences compared to baseline, at 6 months, the COVID-19 group showed an improvement in RV global systolic function, whereas the control group exhibited a decline (∆FAC 1.81 ± 12.03% vs. −0.96 ± 12.09%, *p* = 0.14). By 12 months, FAC continued to decline in the control group, while the COVID-19 group maintained relatively stable values (∆FAC −4.33 ± 12.06% vs. 0.35 ± 12.71%, *p* = 0.01) (Figure 2A).

A repeated-measures ANOVA was conducted to examine the effect of time and COVID-19 status on TAPSE. Time had no significant effect (*p* = 0.34), indicating that TAPSE values remained stable. The time and COVID-19 interaction was not significant (*p* = 0.31), suggesting similar patterns of change across groups. COVID-19 had no significant effect on overall scores (*p* = 0.73). These findings suggest that TAPSE values did not change over time and were not influenced by COVID-19 status.

Examining TAPSE changes from baseline, we observed an initial decline at 6 months in the control group, whereas the COVID-19 group remained relatively stable (∆TAPSE −0.51 ± 4.08 mm vs. 0.10 ± 3.83 mm, *p* = 0.37). By 12 months, values showed numerical improvement in both groups compared to baseline (∆TAPSE 0.42 ± 4.39 mm in the control group and 0.10 ± 4.56 mm in the COVID-19 group, *p* = 0.64). However, these changes were not statistically significant, reinforcing that TAPSE values remained stable over time and were not significantly influenced by COVID-19 status (Figure 2B).

A repeated-measures ANOVA was conducted to examine the effect of time and COVID-19 status on RVFWLS. No significant effect of time was found (*p* = 0.44). No significant interaction was observed between time and COVID-19 (*p* = 0.10); therefore, the patterns of change over time did not differ between groups. COVID-19 had no significant effect on overall RVFWLS values (*p* = 0.92). These findings indicate that RVFWLS values remained stable over time and were not influenced by COVID-19 status (Figure 2C).

However, when comparing RVFWLS changes from baseline, COVID-19 patients showed a numerical improvement at 6 months (ΔRVFWLS −1.77 ± 4.18%), while the control group exhibited a slight decline (ΔRVFWLS 0.34 ± 7.79%). This trend persisted at 12 months, with RVFWLS values of 0.54 ± 8.57% in the control group and −1.41 ± 4.35% in the COVID-19 group. Despite these observed differences, the changes did not reach statistical significance.

A repeated-measures ANOVA was conducted to examine the effect of time and COVID-19 status on RV Tei Index scores. Time had a significant effect (*p* = 0.01), indicating changes in the RV Tei Index over time. The time and COVID-19 interaction was not significant (*p* = 0.10), meaning the pattern of change over time was similar between groups. COVID-19 did not have a significant effect on overall scores (*p* = 0.17). A significant quadratic trend over time was found (*p* = 0.01). These results suggest that RV Tei Index scores change over time, but COVID-19 status does not significantly impact these changes (Figure 2D).

To further explore these time-related changes, we compared RV Tei Index values at 6 months and 12 months to baseline. The COVID-19 group showed a significant improvement at 6 months (ΔRV Tei Index −0.05 ± 0.18 vs. 0.00 ± 0.20, *p* = 0.04) and at 12 months (ΔRV Tei Index −0.01 ± 0.24 vs. 0.05 ± 0.17, *p* = 0.02) compared to baseline.

### 3.5. Correlations Between Cardiac Biomarkers and Echocardiographic Parameters

At baseline, a very weak negative correlation was observed between TnI and LV EF (ρ = −0.081, *p* = 0.25). Similarly, there was a weak positive correlation between TnI and LV GLS (ρ = 0.127, *p* = 0.11), suggesting a slight increase in LV GLS values with rising TnI levels, but this, too, was not statistically significant. Higher TnI levels were significantly associated with worse LV Tei Index at baseline (ρ = 0.327, *p* < 0.01). This association weakened but remained statistically significant at 6 months (ρ = 0.157, *p* = 0.04) and disappeared by 12 months.

There was a weak positive correlation between TnI and RVFWLS at baseline (ρ = 0.044, *p* = 0.57) and at 6 months (ρ = 0.156, *p* = 0.07), but neither was statistically significant. By 12 months, this positive correlation, though still weak, became statistically significant (ρ = 0.224, *p* = 0.01). TnI showed a very weak positive correlation with the RV Tei Index at baseline (ρ = 0.099, *p* = 0.17), which was not statistically significant. This relationship became statistically significant at 6 months (ρ = 0.174, *p* = 0.02) but was no longer significant at 12 months (ρ = 0.049, *p* = 0.54).

There was a significant but weak positive correlation between NT-proBNP levels and LV GLS values at baseline (ρ = 0.202, *p* = 0.01) and during follow-up (6 months: ρ = 0.196, *p* = 0.02; 12 months: ρ = 0.230, *p* = 0.01). A positive correlation between NT-proBNP and RVFWLS was observed only at baseline (ρ = 0.167, *p* = 0.03).

Patients with higher NT-proBNP levels also had larger LAVI, with a significant positive correlation found at baseline (ρ = 0.264, *p* < 0.01) and during follow-up (6 months: ρ = 0.197, *p* = 0.01; 12 months: ρ = 0.194, *p* = 0.01). Similarly, NT-proBNP levels were positively correlated with LV filling pressures at baseline (ρ = 0.264, *p* < 0.01) and during follow-up (6 months: ρ = 0.197, *p* = 0.01; 12 months: ρ = 0.194, *p* = 0.01).

## 4. Discussion

In this study, we evaluated the longitudinal changes in left and right ventricular function in CKD patients with and without prior COVID-19 infection over a 12-month period. Our findings suggest that COVID-19 status influenced the trajectory of certain echocardiographic parameters but did not have a significant independent effect on overall cardiac function.

LV EF showed a significant effect of time, with distinct trajectories between groups. This suggests that, in contrast to the expected LV dysfunction in CKD patients, individuals with prior COVID-19 may undergo a different remodeling process, possibly influenced by inflammatory or compensatory mechanisms in the post-infection phase. However, the overall between-group differences in LV EF were not statistically significant, reinforcing the need for further investigation into potential mechanisms. Similarly, the LV Tei Index was significantly affected by time and COVID-19 status, with the COVID-19 group showing greater improvements at 6 and 12 months compared to controls. This suggests that COVID-19 may have influenced global LV performance, leading to transient functional adaptations.

For RV function, FAC demonstrated significant changes over time, with the control group exhibiting a progressive decline, whereas the COVID-19 group maintained more stable values. At 12 months, the between-group difference in FAC reached statistical significance, suggesting a possible compensatory effect in the COVID-19 group. Although the overall interaction effect was not significant, this finding warrants further exploration. Conversely, TAPSE and RVFWLS remained stable over time, with no significant changes observed in either group. This suggests that while LV function exhibited notable temporal changes, RV function, as measured by TAPSE and RVFWLS, was less affected over the study period. Notably, the RV Tei Index demonstrated significant time-related changes, with the COVID-19 group showing greater improvements at both 6 and 12 months. While the interaction effect was not significant, these findings suggest potential differences in RV global performance over time.

LV dysfunction has been reported in 5.4% to 40% of COVID-19 cases [18,19]. In a study including 40 COVID-19 patients compared to 40 healthy subjects, although there were no significant differences in LV EF between the two groups, the myocardial performance index was significantly more impaired in the COVID-19 group (0.56 ± 0.09 versus 0.41 ± 0.06). After approximately 2 weeks, a significant improvement was observed (0.44 ± 0.07 versus 0.56 ± 0.09) [20]. Our study yielded similar results, showing significant baseline differences between the groups in terms of the LV Tei index. Moreover, the LV Tei index had a sustained improvement during follow-up. The decline in LV EF at 12 months may be attributed to the progression of uremic cardiomyopathy. However, the literature also reports a reduction in LV EF one year post-COVID-19 in the general population [4].

The development of new echocardiographic modalities, such as speckle-tracking, provides a detailed assessment of heart function, detecting not only clinical but subclinical myocardial dysfunction. LV GLS was impaired among COVID-19 patients, regardless of EF value or preexisting CV diseases, and even in mild forms of the disease. When evaluating the LV GLS, approximately one-third of patients suffer subclinical myocardial dysfunction secondary to COVID-19 [21,22,23]. Impaired LV GLS was associated with worse CV outcomes, suggesting the importance of adequate follow-up of COVID-19 patients after discharge due to a higher risk of adverse outcomes [24]. In our study COVID-19 patients did have a slightly lower LV GLS value, though not reaching statistical significance. On the other hand, LV GLS has significantly lower values in patients with kidney impairment compared to those with normal kidney function and is an independent predictor for both MACE and all-cause mortality, with various cut-off values established depending on the population studied: −15% for dialysis patients and −17.7% for patients with CKD who are not on dialysis [25,26]. Data on serial speckle-tracking echocardiographic evaluations in COVID-19 patients is scarce. One study that evaluated patients at a median time of 130.35  ±  76.06 days after COVID-19 diagnosis reported a reduction in mean longitudinal strain for the basal segment in the COVID-19 group [27]. However, a recent study showed that LV dysfunction evaluated by GLS found in one-third of recovered COVID-19 patients mildly improves during the first year [22].

Kidney dysfunction is also associated with diastolic dysfunction, showing a significant inverse relationship: as the estimated glomerular filtration rate decreases, LV diastolic dysfunction worsens [28,29]. Our study showed no difference in the E/e′ ratio; however, although no significant differences were found at baseline, COVID-19 patients had a significantly larger LAVI at 6 months and one-year evaluation. This may be explained by persistent low-grade atrial inflammation post-COVID-19, altered atrial compliance due to endothelial dysfunction, and potential long-term effects of altered pulmonary hemodynamics post-infection [30]. Moreover, both groups had enlarged LAVi, and this is most probably explained by the association of CKD [31]. To our knowledge, this is the first study to demonstrate a statistically significant increase in LAVi in patients with COVID-19.

The normal hemodynamics of the RV are characterized by low resistance. A sudden increase in these resistances under certain pathological conditions, such as acute respiratory distress syndrome, will lead to RV dilation and dysfunction, as the RV is anatomically defined by a thin free wall [32]. Echocardiographic evaluations of patients during the acute infectious episode concluded that patients more frequently presented with RV dysfunction and dilation [18]. The reported prevalence of longitudinal RV dysfunction, evaluated by TAPSE and tricuspid S′, in hospitalized patients with SARS-CoV-2 infection ranges from 10 to 25% [5,33]. When other parameters of global RV dysfunction were evaluated, including the FAC or Tei Index, the prevalence of RV dysfunction increased to 72% [34]. However, this increased prevalence was reported in critically ill patients. In our study, the prevalence of RV dysfunction in COVID-19 patients was 30.6%.

Recent studies indicate an increased prevalence of RV dysfunction in dialysis patients, along with its association with a higher risk of developing arrhythmias and an increased risk of mortality. Advanced age, chronic inflammatory status, poor nutritional status, and greater interdialytic weight gain are contributing factors to the onset of RV dysfunction. In patients with heart failure, RV dysfunction measured by TAPSE was associated with the presence of CKD, suggesting a correlation between the two [35]. The interplay between the RV and kidney function is further sustained by a study that evaluated patients without CV disease and found that mild reduction in the glomerular filtration rate was associated with subclinical RV remodeling, more specifically smaller RV volumes, probably due to impaired relaxation and fibrosis [36]. Although the basal diameter of the RV and FAC may not differ between dialysis patients and those with normal kidney function, the RVFWLS is significantly more impaired in dialysis patients [37].

In a study that included patients without prior heart disease, although no significant RV systolic dysfunction was seen in the first year after COVID-19 infection, there were some changes in echocardiographic parameters: while TAPSE, RVFWLS, maximum velocity of the tricuspid regurgitation and right atrial volume had gradually decreased at the one year evaluation, other parameters, such as RV FAC, pulmonary valve flow acceleration time, early diastolic mitral annulus velocity, and parameters of left atrial function improved [4]. We reported similar results, with an improvement in FAC at 12 months and a decrease in RVFWLS. TAPSE and RV Tei index also improved from 6 months. Identifying these alterations is of valuable prognostic importance, given that a decrease as small as 1 mm in TAPSE values was associated with a mortality increase as high as 20% [38].

According to available data on hemodialysis patients, the NT-proBNP and Troponin I biomarkers present conflicting results, either increasing or decreasing, due to various factors, such as the dialysis itself, the ultrafiltration, hemoconcentration, the timing of sampling (predialysis, postdialysis), malnutrition, types of dialyzers (high/low flux), degree of inflammation, overhydration, dialysis vintage, dialysis session period, or exposure to microemboli of air that are present during dialysis, or some other variable within the individual patient that affects these changes [39,40,41,42,43]. All the dialysis patients from our study belong to dialysis centers that follow the same protocols, and all the blood samples were collected on the second day after a dialysis session during the short interdialytic interval.

Our results highlight that while COVID-19 did not independently alter absolute ventricular function, it influenced the pattern of change over time. This could reflect differences in post-COVID-19 inflammatory resolution, myocardial adaptation, or hemodynamic responses in CKD patients. The preserved or improved indices in the COVID-19 group, particularly for LV Tei Index, FAC, and RV Tei Index, may suggest a temporary adaptive phase following infection, potentially modulated by post-COVID-19 cardiac remodeling.

Limitations of our study refer to the determination of serum biomarkers in patients with CKD. Both BNP and NT-pro-BNP levels are influenced by renal impairment, and CKD patients exhibit significantly elevated circulating levels of these natriuretic peptides [44]. Evidence suggests that reduced renal clearance substantially impacts NT-proBNP levels more than BNP [45]. Despite its greater reliance on renal clearance, NT-proBNP has demonstrated superior accuracy in predicting systolic heart failure when higher cut-off values are applied, such as cutoffs of 300 pg/mL and 4502 pg/mL, respectively [46]. However, optimal cut-off thresholds for BNP and NT-proBNP in the context of significant renal failure have yet to be determined.

Selection bias was minimized by including all consecutive eligible CKD patients from the participating centers. To minimize misclassification bias, prior COVID-19 infection was confirmed through documented RT-PCR results and clinical history. Echocardiographic measurements were performed using standardized protocols by trained cardiologists, and intra- and inter-observer variability was assessed in a random subset of patients. To minimize variability due to volume status, echocardiography was performed on the second day after a dialysis session, during the short interdialytic interval.

## 5. Conclusions

This study demonstrates that in CKD patients, prior COVID-19 infection influenced the trajectory of LV and RV function over 12 months without significantly altering overall cardiac function. While LV EF, LV Tei Index, FAC, and RV Tei Index showed differential changes between groups, other RV parameters (TAPSE and RVFWLS) remained stable. These findings suggest potential transient myocardial adaptations following COVID-19, which warrant further investigation. A significant increase in LAVi was noted in patients with COVID-19.

This study is the first to assess serial echocardiographic parameters in a CKD population affected by COVID-19 over a one-year follow-up period. While echocardiographic parameters were influenced by the presence of kidney disease, our findings are consistent with other echocardiographic reports in the literature. Continuous echocardiographic monitoring in CKD patients, particularly those with prior COVID-19, may help identify early functional changes and guide appropriate clinical management strategies.

## Figures and Tables

**Table 1:** Demographics of the study population.

All Patients *n* = 206	Age (Years)	Male (%)	Smoker (%)	HTN (%)	DM (%)	Afib (%)	HF (%)	CAD(%)	Pulmonary Disease (%)
COVID-19 *n* = 121	58.87 ± 15.27	52.1	6.6	82.6	33.1	20.7	47.9	32.2	6.6
Control *n* = 85	54.25 ± 15.53	55.3	16.5	75.3	17.6	11.8	29.4	15.3	5.9
	*p* = 0.04	*p* = 0.64	*p* = 0.02	*p* = 0.19	*p* = 0.01	*p* = 0.09	*p* = 0.01	*p* = 0.01	*p* = 0.83
HD patients *n* = 134									
COVID-19 *n* = 79	62.72 ± 14.45	48.1	7.6	82.3	38.0	27.8	55.7	39.2	8.9
Control *n* = 55	59.93 ± 62.72	50.9	21.8	76.4	20.0	18.2	38.2	20.0	5.5
	*p* = 0.22	*p* = 0.74	*p* = 0.01	*p* = 0.40	*p* = 0.02	*p* = 0.19	*p* = 0.04	*p* = 0.01	*p* = 0.46

HTN—arterial hypertension; DM—diabetes mellitus; Afib—atrial fibrillation; HF—heart failure; CAD—coronary artery disease.

**Table 2:** Baseline echocardiographic parameters in all patients.

Echocardiographic Parameter	COVID-19 Group N= 121	CONTROL Group N = 85	*p* Value
	Left ventricle		
IVS (mm)	12.14 ± 2.22	12.48 ± 2.29	0.23
LVPW (mm)	11.71 ± 2.18	12.47 ± 2.16	0.01
LVEDD (mm)	46.73 ± 7.36	44.69 ± 5.92	0.03
LVESD (mm)	30.77 ± 7.25	27.01 ± 5.26	<0.01
LV EDV (ml)	112.03 ± 49.88	92.50 ± 33.57	0.01
LV ESV (ml)	51.37 ± 34.74	39.72 ± 18.96	0.01
LV EF (%)	55.52 ± 10.15	57.36 ± 8.77	0.19
	57 (IQR 50.75–62.25)	58 (IQR 53–63)	
LV GLS (%)	−16.86 ± 4.44	−17.72 ± 2.81	0.12
	−18.00 (IQR (−20.00, −16.00)	−18.5 (IQR −20.875, −16.125)	
LV Tei index	0.60 ± 0.16	0.50 ± 0.12	<0.01
	Right ventricle		
TAPSE (mm)	21.61 ± 4.81	22.17 ± 4.64	0.56
	21 (IQR 18–24)	21 (IQR 18–24)	
FAC (%)	40.56 ± 10.82	45.02 ±8.06	0.01
RVFWLS (%)	−18.01 ± 5.27	−20.46 ± 2.93	0.01
	−18 (IQR −21, −15)	−21.00 (IQR −23.50, −18.50)	
Tricuspid S’ velocity (cm/s)	13.12 ± 2.91	12.76 ± 2.93	0.38
RV Tei Index	0.60 ± 0.17	0.54 ± 0.16	0.01
PAAT (ms)	96.10 ± 26.17	100.40 ± 25.56	0.24
TR Vmax (m/s)	2.16 ± 0.72	2.12 ± 0.52	0.65
PASP (mmHg)	26.31 ± 14.30	23.74 ± 10.33	0.37
Proximal RVOT (mm)	34.57 ± 5.00	32.94 ± 4.49	0.01
RV basal diameter (mm)	36.58 ± 7.18	33.72 ± 6.03	0.01
RV mid diameter (mm)	29.28 ± 7.26	25.31 ± 5.95	<0.01
RV longitudinal diameter mm)	69.91 ± 10.18	69.76 ± 8.80	0.91
RV free wall thickness (mm)	5.96 ± 1.44	5.39 ± 1.51	0.01
Inferior vena cava (mm)	16.29 ± 3.41	14.79 ± 3.03	0.01
	Diastolic function		
LAVI (mL/m^2^)	43.17 ± 17.84	39.07 ± 16.22	0.06
DTE (ms)	196.85 ± 51.41	195.33 ± 53.77	0.84
E-wave velocity (cm/s)	86.08 ± 29.57	80.10 ± 26.20	0.18
A-wave velocity (cm/s)	87.26 ± 24.25	87.64 ± 28.51	0.62
E/A	0.94 ± 0.37	0.92 ± 0.41	0.56
medial e′ velocity (cm/s)	6.98 ± 2.08	6.97 ± 2.28	0.84
lateral e′ velocity (cm/s)	9.69 ± 3.17	9.73 ± 3.21	0.92
E/e′	11.19 ± 4.29	10.68 ± 4.89	0.24

Abbreviations: IVS—interventricular septum; LVPW—left ventricular posterior wall; LVEDD—left ventricular end-diastolic diameter; LVESD-left ventricular end-systolic diameter; LV EDV—left ventricular end-diastolic volume; LV ESV—left ventricular end-systolic volume; LV EF—left ventricular ejection fraction; LV GLS—left ventricular global longitudinal strain; TAPSE—Tricuspid annular longitudinal excursion; FAC—fractional area change; RVFWLS—right ventricular free wall longitudinal strain; PAAT—pulmonary artery acceleration time; TR Vmax—maximal tricuspid regurgitation velocity; PASP—pulmonary artery systolic pressure; RVOT—right ventricular outflow tract; LAVI—indexed left atrial volume; DTE—early mitral flow deceleration times; RV—right ventricular; IQR—interquartile range.

**Table 3:** Serial biomarker evaluations.

Biomarkers	COVID-19 Group N = 88	CONTROL Group N = 75	*p* Value
	Baseline		
TnI (pg/mL)	53.02 ± 60.42	14.15 ± 14.70	0.01
NT-proBNP (ng/mL)	4.69 ± 6.16	2.69 ± 3.11	0.37
	6 months		
TnI (pg/mL)	37.82 ± 45.44	12.86 ± 15.17	<0.01
NT-proBNP (ng/mL)	10.61 ± 37.72	5.56 ± 21.81	<0.01
	12 months		
TnI (pg/mL)	15.84 ± 15.26	17.00 ± 16.19	0.21
NT-proBNP (ng/mL)	7.39 ± 32.95	3.74 ± 21.07	0.01

TnI = troponin I, NT-proBNP = N-terminal pro–B-type natriuretic peptide.

**Table 4:** Baseline LV and RV systolic function echocardiographic parameters in survivors only.

Echocardiographic Parameter	COVID-19 Group N = 88	CONTROL Group N = 75	*p* Value
LVEF (%)	55.51 ± 10.23	57.74 ± 8.87	0.11
	57 (IQR 50.50–63.50)	59 (IQR 53.85–64.15)	
LV GLS (%)	−17.18 ± 4.13	−18.04 ± 2.69	0.14
	−18.00 (IQR (−19.94, −16.06)	−19.00 (IQR −21.00, −17.00)	
LV Tei index	0.59 ± 0.16	0.50 ± 0.11	<0.01
TAPSE (mm)	22.40 ± 4.48	22.69 ± 4.49	0.86
	21.5 (IQR 18.5–24.5)	22 (IQR 18.5–25.5)	
FAC (%)	41.14 ± 10.90	45.38 ± 8.08	0.01
RVFWLS (%)	−18.80 ± 4.47	−20.64 ± 2.94	0.01
	−18.60 (IQR −20.60,−16.60)	−21.00 (IQR −23.50,−18.50)	
Tricuspid S’ velocity (cm/s)	13.51 ± 2.82	12.88 ± 2.77	0.16
RV Tei Index	0.59 ± 0.18	0.53 ± 0.15	0.01

LV EF—left ventricular ejection fraction; LV GLS—left ventricular global longitudinal strain; TAPSE—tricuspid annular longitudinal excursion; FAC—fractional area change; RVFWLS—right ventricular free wall longitudinal strain; RV—right ventricular; IQR—interquartile range.

**Table 5:** Serial echocardiographic evaluations at 6 months and 12 months.

Echocardiographic Parameter	COVID-19 Group (N = 88) 6 Months	CONTROL Group (N = 75) 6 Months	*p* Value	COVID-19 Group (N = 88) 12 Months	CONTROL Group (N = 75) 12 Months	*p* Value
LVEF (%)	57.07 ± 9.77	55.34 ± 7.70	0.16	55.11 ± 9.12	54.85 ± 7.70	0.50
	57.5 (IQR 51.5–63.50)	54.5 (IQR 50.05–58.95)		56.45 (IQR 50.95–61.95)	55 (IQR 50.35–59.65)	
LV GLS (%)	−18.47 ± 3.79	−18.61 ± 2.93	0.82	−16.76 ± 3.62	−17.53 ± 3.04	0.36
LV Tei index	0.59 ± 0.15	0.49 ± 0.10	0.01	0.51 ± 0.13	0.53 ± 0.11	0.29
TAPSE (mm)	22.51 ± 4.02	22.20 ± 4.21	0.64	22.51± 4.28	23.12 ± 4.01	0.35
FAC (%)	42.95 ± 9.92	44.53 ±9.28	0.29	41.49 ± 10.08	41.05 ±8.94	0.76
RVFWLS (%)	−21.03 ± 3.73	−20.15 ± 7.72	0.67	−20.41 ± 4.44	−20.01 ± 8.19	0.65
	−21.00 (IQR −23.08, −18.93)	−21.00 (IQR −22.5, −19.5)		−21 (IQR −23.5, −18.5	−21 (IQR −23.25, −18.75)	
Tricuspid S’ velocity (cm/s)	12.78 ± 2.94	12.70 ± 3.27	0.86	12.30 ± 2.95	12.76 ± 3.17	0.34
RV Tei Index	0.54 ± 0.14	0.53 ± 0.14	0.38	0.58 ± 0.19	0.58 ± 0.16	0.87
PAAT (ms)	102.18 ± 26.00	110.16 ± 24.21	0.04	98.27 ± 21.62	102.29 ± 22.91	0.22
Proximal RVOT (mm)	33.73 ± 4.31	31.58 ± 4.23	0.01	33.06 ± 4.18	31.24 ± 4.90	0.01
LAVI (ml/m^2^)	40.73 ± 14.65	34.71 ± 12.64	0.01	40.26 ± 20.71	33.40 ± 13.25	0.01
E/e’	11.15 ± 3.51	10.38 ± 4.15	0.07	11.38 ± 4.16	10.78 ± 4.48	0.26

LV EF—left ventricular ejection fraction; LV GLS—left ventricular global longitudinal strain; TAPSE—Tricuspid annular longitudinal excursion; FAC—fractional area change; RVFWLS—right ventricular free wall longitudinal strain; PAAT—pulmonary artery acceleration time; RVOT—right ventricular outflow tract; LAVI—indexed left atrial volume; RV—right ventricular; IQR—interquartile range.

## Data Availability

The data underlying this article will be shared on reasonable request by the corresponding author.
